# Constructing and Characterizing Bacteriophage Libraries for Phage Therapy of Human Infections

**DOI:** 10.3389/fmicb.2019.02537

**Published:** 2019-11-12

**Authors:** Shelley B. Gibson, Sabrina I. Green, Carmen Gu Liu, Keiko C. Salazar, Justin R. Clark, Austen L. Terwilliger, Heidi B. Kaplan, Anthony W. Maresso, Barbara W. Trautner, Robert F. Ramig

**Affiliations:** ^1^Department of Molecular Virology and Microbiology, Baylor College of Medicine, Houston, TX, United States; ^2^Department of Microbiology and Molecular Genetics, McGovern Medical School, University of Texas Health Science Center at Houston, Houston, TX, United States; ^3^Center for Innovations in Quality, Effectiveness and Safety, Michael E. DeBakey VA Medical Center, Houston, TX, United States; ^4^Department of Medicine, Baylor College of Medicine, Houston, TX, United States

**Keywords:** phage libraries, phage therapy, host range, phage characteristics, killing spectrum, human infection

## Abstract

Phage therapy requires libraries of well-characterized phages. Here we describe the generation of phage libraries for three target species: *Escherichia coli*, *Pseudomonas aeruginosa*, and *Enterobacter cloacae*. The basic phage characteristics on the isolation host, sequence analysis, growth properties, and host range and virulence on a number of contemporary clinical isolates are presented. This information is required before phages can be added to a phage library for potential human use or sharing between laboratories for use in compassionate use protocols in humans under eIND (emergency investigational new drug). Clinical scenarios in which these phages can potentially be used are discussed. The phages presented here are currently being characterized in animal models and are available for eINDs.

## Introduction

The crisis in clinical care imposed by the increasing resistance of bacterial infections to antibiotics threatens to return clinical practice to the pre-antibiotic era (Boucher et al., 2009; Centers for Disease Control [CDC], 2013; Bassetti et al., 2017; World Health Organization [WHO], 2017). The situation is particularly acute for infections caused by Gram-negative pathogens for which few new antibiotics are in the pipeline. The bacterial “mutagenic tetrasect” (mutation, transformation, transduction, and conjugation) is responsible for the rapid evolution of bacteria and suggests that bacteria are so flexible in their ability to adapt that production of antibiotics by pharmaceutical companies, will never be able to keep up with the evolution of resistance against that drug. Fortunately, a natural alternative to conventional chemical antibiotics (Ghosh et al., 2018; Stearns, 2019) exists in the form of bacteriophages (phages). Thus, phages can evolve to efficiently target specific bacteria and have been used to treat complex drug-resistant bacterial infections in a procedure termed phage therapy (Ghosh et al., 2018).

Although phage therapy has great potential as a treatment for antibiotic resistant infections, it is not without problems. Phage have a similar mutation rate to bacteria; the organisms reproduce so rapidly that the high numbers lead to very large mutant populations on which selection can operate to enrich selected phenotypes. Host range expansion by evolution and selection has been achieved (Burrowes, 2011; Mapes et al., 2016; Burrowes et al., 2019) and is extremely rapid (unpublished data). In addition, phage ecologists have estimated the total number of phages on Earth to be greater than 10^31^ (Suttle, 2005). This suggests that the environment is a plentiful resource for new phages; indeed, environmental phages to drug-resistant and pandemic *Escherichia coli* that have excellent efficacy in animal models of infection have been discovered, characterized, and tested in as short a time period as 14 days (Green et al., 2017).

Phages were discovered over a century ago, and phage therapy has a long history (Debarbieux et al., 2018; Gelman et al., 2018). However, the advent of chemical antibiotics led to virtual abandonment of phage therapy in most of the world, whereas in the countries of Eastern Europe phage therapy was continuously pursued (Chanishvili, 2016; Myelnikov, 2018). As the development of antibiotic resistance has grown, the interest in phage has been rekindled (Kutter et al., 2015). Studies with compassionate use investigational new drug (IND) have generated considerable excitement for use of phage therapy in human subjects (Wright et al., 2009; Schooley et al., 2017; Chan et al., 2018; Aslam et al., 2019). Recently the application of phage therapy to human infections was reviewed (El Haddad et al., 2018), and the majority of the studies analyzed showed efficacy (87%) and safety (67%), however only a few of the studies examined the development of phage-resistant bacteria during therapy. Bacteria become resistant to phage infection (phage-resistant) (Labrie et al., 2010), through mutational changes just as they become antibiotic-resistant upon treatment with antibiotics. The problem of phage-resistance is often overcome by [i] the use of single broad host range phages, [ii] phages for which development of phage-resistance carries high bacterial fitness costs (Chan et al., 2016; unpublished data), or [iii] mixtures (cocktails) of phages (generally recognizing different bacterial surface receptors). Others have argued that phage resistance is not a problem in phage therapy because phage resistant bacteria often have fitness defects and new environmental phages active on the resistant host can be isolated (Ormala and Jalasvuori, 2013). Indeed it is likely that phages capable of infecting a resistant host can be isolated from the environment or evolved in the laboratory (Mapes et al., 2016, unpublished data). However, these operations are time consuming and best avoided. In addition, not all phage resistant hosts were found to have fitness defects, so that they could persist in the patient (Wei et al., 2010, our unpublished data).

The development of phages for use against human infections has been described as following two pipelines (Pirnay et al., 2011). The “*prêt à porter*” (ready-to-wear) is a method, in which a medicinal product of a single broad host range phage is developed and undergoes safety and efficacy testing. This is time consuming and costly but yields products that can be licensed by regulatory agencies. In contrast, in the “*sur-mesure*” (custom made) method many phages are isolated and characterized and can be combined as appropriate to treat the infection. This method is flexible, inexpensive, and can rapidly respond to infections with phage- or antibiotic- resistant bacteria. However, *sur-mesure* approaches cannot currently be licensed, but therapeutic use of phage produced through this approach is under active discussion (Debarbieux et al., 2016; Pirnay et al., 2018). We have chosen the latter approach in which [i] libraries of well-characterized phages are generated and stored, [ii] as the clinical laboratory is assessing the antibiotic-resistance of the bacterial isolate (∼48 h), it is also tested for sensitivity to phages from the library (<48 h), [iii] phages to which the clinical isolate is sensitive are selected for mono-phage-therapy or used to construct cocktails containing several individual phages. Two therapeutic options are available: the patient can be treated with the phage alone, or phage plus antibiotic.

Here, we describe the preparation of well-characterized libraries of *E. coli*-specific, *Pseudomonas aeruginosa*-specific, and *Enterobacter cloacae*-specific phages for use in a *sur-mesure* approach to phage therapy, which will ultimately result in a therapeutic that is personalized to the specific infection of the individual patient. We provide information on the phages including: descriptions of their sources, their efficacy against a panel of clinical strains, some basic infection characteristics (burst size and absorption rates), and their DNA sequence and annotation to determine if they harbor lysogenic, antibiotic resistance or toxin genes and their morphologic description; providing the means to select high quality phage(s) for use in therapy. Also described are the clinical scenarios for which these phage libraries have been developed, as their proposed use shaped the development of the libraries.

## Materials and Methods

### Bacterial Strains

The laboratory strains used were *E. coli* (MG1655) and *P. aeruginosa* (PAO1 and BWT111). A collection of 13 *E. coli* ST131 strains (see Supplementary Figures S1–S3) was obtained from Dr. Jim Johnson (University of Minnesota). Two strains of *E. cloacae* were isolated from a phage therapy candidate with an infected hip prosthesis. One isolate was from a wound swab and other from the fluid exudate of the wound (collected at different times). De-identified clinical isolates of *E. coli*, *P. aeruginosa*, and *E. cloacae* and their antibiotic susceptibility data were obtained from the clinical microbiology laboratory at the Houston Veterans Administration Hospital or Baylor St. Luke’s Hospital. Collection of de-identified clinical isolates was approved by the Baylor College of Medicine Institutional Review Board (IRB). An isolated colony of each clinical isolate was streaked on LB agar and grown overnight. A single colony from the LB plate was grown overnight in LB medium, diluted 1:10 into LB medium containing 15% glycerol, and frozen at −80°C. In cases where clinical isolates appeared to be mixed, the desired species was isolated from differential plates.

### Phages

**FIGURE 1:**
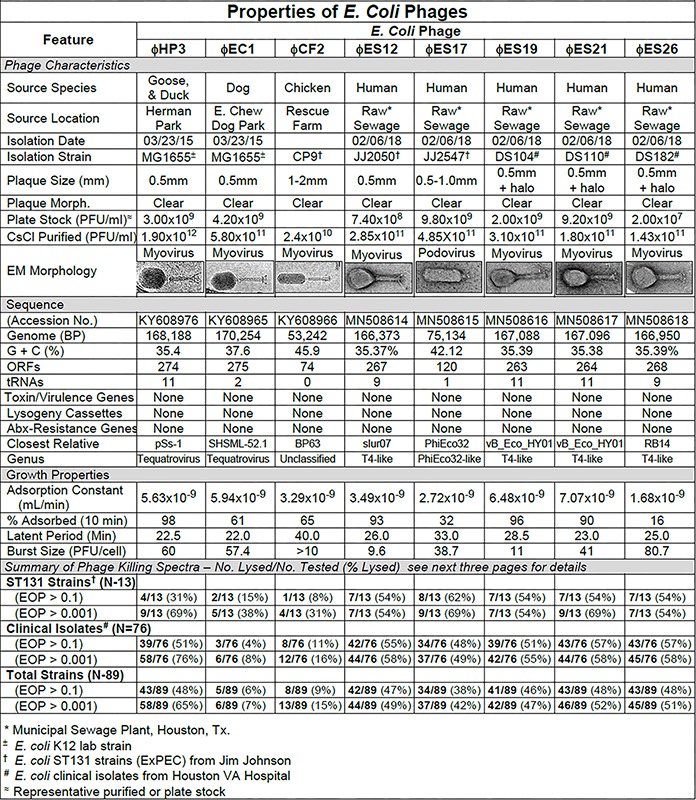
Summary of characterization of *Escherichia coli* phages. The characteristics, DNA sequences, growth properties and a summary of phage killing spectra are presented for each phage.

Four *P. aeruginosa*-specific phages ϕKMV, ϕPA2, ϕPaer4, and ϕE2005-24-39 (hereafter called ϕE2005) were previously described (Mapes et al., 2016). These were the only *Pseudomonas* wild type phages used in this work. All *E. coli*-specific and *E. cloacae* phages used here were isolated from environmental samples (see Figures 1, 4A,B, 7 for source species) by plaque assay. Fecal samples were suspended to ∼50% (w/v) in PBS, shaken, and centrifuged to remove debris. The supernatant was filtered through a 0.22 micron filter, and 0.1 ml was plated with 0.8% LB top agar containing 100 μl of an overnight culture of the desired isolation strain. After overnight incubation, well-isolated plaques were picked into 1.0 ml phage storage buffer (Mapes et al., 2016), allowed to sit overnight for phage diffusion at 4°C, and 0.5 ml of suspended phage was used to prepare plate stocks using the isolation strain as host. Plate stocks were harvested and stored at 4°C.

### Host Range Determination/Efficiency of Killing (Virulence)

**FIGURE 2:**
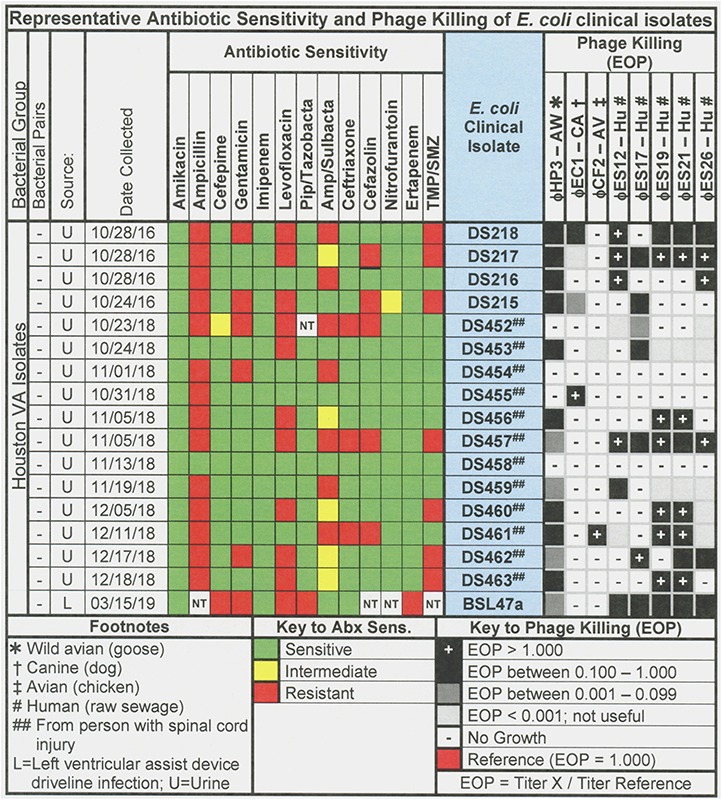
Representative data for antibiotic sensitivity and phage killing (EOP) of clinical isolates of *E. coli*. Shown are the properties of the *E. coli* clinical isolates on the left, including: source, date of isolation and antibiotic sensitivity data (VITEK2). On the right are shown the killing spectra of the phages on the individual *E. coli* clinical isolates. The keys to antibiotic sensitivity and phage killing (EOP) are shown at the bottom of the figure.

To determine phage host range a spot titration protocol was used that allowed us to determine both host range and relative phage killing (EOP). Five microliters of serial 10-fold dilutions of a CsCl purified phage stock (10^10^–10^12^ pfu/ml) were spotted on freshly seeded lawns of control, isolation, or clinical strains. The host range and titer were determined by formation of individual plaques within the area of the spot at terminal dilution. This avoided false positives by determining host range at dilutions where phenomena such as lysis from without (Abedon, 2011) or complementation between defective phages would not be expected. Phage virulence was determined as the efficiency of plating (EOP) (Mirzaei and Nilsson, 2015). EOP was calculated by dividing the titer of the phage at the terminal dilution on the test strain by the titer of the same phage on its isolation strain. On this basis, phages were classified as highly virulent (0.1 < EOP > 1.00), moderately virulent (0.001 < EOP < 0.099), avirulent but active (EOP < 0.001), or avirulent (no plaques detected – see Figures 2, 5, 8).

### Host Range Expansion (HRE)

*Pseudomonas aeruginosa*-specific phages were subjected to the HRE protocol as described (Burrowes, 2011; Mapes et al., 2016; Burrowes et al., 2019). Briefly, in a 96 well plate, different host strains were placed in each of the eight rows at a dilution of 1:1000 of overnight culture. Serial 10-fold dilutions of phage (a single phage or a phage mixture) were placed in the 12 columns and the plate was incubated (37°C) with shaking (225 RPM) for 18 h. Following incubation, for each bacterial strain (row) the supernatant from the well with complete lysis and the adjacent well with higher phage dilution (partial lysis) were combined, into a single tube with the corresponding complete and partial lysis wells of the other bacterial strains. The pooled lysate was treated with CHCl_3_ and filtered through a 0.22 micron filter. The filtered lysate was the yield of the 1^st^ cycle of HRE. This filtered lysate was serially diluted 10-fold, and the experiment was repeated using the pooled lysate as the phage and the same bacterial strains as host for cycle 2. The HRE was repeated up to 30 cycles, and yielded a mixture of phages that had replicated on at least one of the host bacterial strains. The heterogeneous mixture of phages in the lysate from any cycle of HRE can be assayed for plaque formers on a host refractory to the parental phage(s), plaques purified, and phage stocks with expanded host range produced (Mapes et al., 2016). The HRE protocol was also successfully applied to *E. coli*-specific phages (data not shown).

The HRE protocol exposes the lysate (including mutants) that arose during a cycle to new bacteria (unevolved) of the strains used in the previous cycle. Some of the mutations may allow phages to infect and replicate on bacterial strains that were previously refractory to phage, thus amplifying the mutant that contained the host range expanding mutation.

### DNA Sequencing and Annotation of Phage Genomes

CsCl purified phages were submitted to the Center for Metagenomics and Microbiome Research at Baylor College of Medicine for DNA extraction, sequencing and assembly as described previously (Green et al., 2017). Briefly, DNA samples were constructed into Illumina paired-end libraries. The libraries had an average final size of 660 bp (including adapter and barcode sequences) and were pooled in equimolar amounts to achieve a final concentration of 10 nM. The library templates were prepared for sequencing on the Illumina MiSeq. After sequencing, the.bcl files were processed through Illumina’s analysis software (CASAVA), which demultiplexes pooled samples and generates sequence reads and base-call confidence values (qualities). The average raw yield per sample was 802 Mbp. For analysis, the adapter sequences were removed, and the sequence was then assembled using SPAdes v3.5.0 (Bankevich et al., 2012) on careful mode, retaining only contigs longer than 1,000 bp and with an average coverage of 1000x or greater. This generated 1–2 contigs per sample, with an average of 74% of the original reads mapping with 100% identity to the final contigs. Genomes were analyzed using both PATRIC’s comprehensive genome analysis service (Wattam et al., 2017) and EDGE Bioinformatic software (Li et al., 2017). Gene calling and genome annotation was performed using PROKKA (version 1.13) (Lo and Chain, 2014), RAST (Zerbino and Birney, 2008; Bankevich et al., 2012), GLIMMER3 (version 3.02) (Peng et al., 2010), and GeneMarkS (version 4.28) (Peng et al., 2012). Figures 1, 4A,B, 7 show ORF predictions from the RAST pipeline and tRNA predictions from ARAGORN (version 1.2.36) (Seemann, 2014). ORFs were searched for virulence and antibiotic resistance genes by using BLAST (Aziz et al., 2008) to compare assembled genomes against the Virulence Factor Database (VFDB) (McNair et al., 2018), the PATRIC virulence factor database (Delcher et al., 2007), the Antibiotic Resistance Gene Database (ARDB) (Besemer, 2001) and the Comprehensive Antibiotic Resistance Database (CARD) (Laslett, 2004). ShortBRED (version 0.9.4M) (Johnson et al., 2008) was used for targeted searches of ORFs for genes in the VFDB, CARD, and the Resfam antibiotic resistance gene database (Chen et al., 2016). Genus was inferred from closest sequenced relatives identified by using BWA-Mem (version 0.7.9) (Mao et al., 2015) to aligning contigs to NCBI’s RefSeq database and by ORF homology using PHAge Search Tool Enhanced Release (PHASTER) (Liu and Pop, 2009). Phage lifestyles were determined by classifying the genomes using PHACTs (McArthur et al., 2013), using PHASTER to predict integrases and attachment sites, and by parsing all versions of the annotated genome for “integrase.” No virulence genes of known toxicity (viral or bacterial) or genes involved in lysogeny were detected. Thus it appears the phages examined here are devoid of any known lysogenic or toxic elements that would preclude their use in phage therapy.

### Phage Growth Parameters

The percentage of phage adsorbed in 10 min and the adsorption constant were determined for each phage on its isolation strain. Burst size and latent period (one-step growth curves) were also determined for each phage on its isolation strain (Kropinski, 2009, 2018).

## Results

### *Escherichia coli* Phages

#### *E. coli* Phage Isolation

Our primary target for *E. coli* phage isolation was extraintestinal pathogenic *E. coli* (ExPEC) of the pandemic sequence type 131 (ST131). ExPEC are commonly associated with bacteremia and urinary tract infections, and the ST131 lineage is characterized by multi-drug resistance and its high frequency of isolation over the past 10 years. Interestingly, a rapid screen of common laboratory *E. coli*-specific phages (T2, T4, T6, T7, λ^vir^) revealed that none of them formed plaques on the ST131 strains tested. As a result we began to isolate phages from the environment, concentrating on avian and canine species which are known reservoirs for *E. coli* ST131 (Johnson et al., 2001, 2017). Our first phage isolates were from mixed goose/duck feces collected at a local park (ϕHP3), canine feces from a dog park (ϕEC1) and chicken feces from a rehabilitation farm (ϕCF2). We subsequently isolated phages from raw sewage collected at a local sewage treatment plant (ϕES series; see Figure 1). All phages were plaque-purified three times prior to use.

#### *E. coli* Phage Characteristics

The phage characteristics are summarized in Figure 1. The phages varied in plaque size, but plaques tended to be small and clear. Some produced halos around the plaques, suggesting enzymatic activity on the surrounding cells. Regardless of the small plaque sizes, reasonable titer plate stocks were obtained, and all phages could be CsCl purified to about 10^11^ pfu/ml. Adsorption curves revealed that the phages ranged widely in adsorption (16–98%). For all the *E. coli* phages, one-step growth curves revealed the latent period was in the range of 22–40 min and burst sizes ranged from 9.6 to 80.7 pfu/cell. Sequence analysis revealed genome sizes ranging from (53,242 to 170,254 bp) with variable G + C content. The number of encoded ORFs and tRNAs identified was also variable. Notably, none of the sequences contained features that would preclude their use in phage therapy, such as genes to establish and maintain lysogeny, produce toxins or virulence factors, or confer antibiotic resistance (Merabishvili et al., 2018; Hyman, 2019). All of the *E. coli* phages had myovirus morphology except for ϕES17 which was a podovirus with an elongated head. Phage ES17 also contained, at marginal statistical significance, an integrase gene when examined with PHACTs and PHASTER software. When colonies were isolated in the presence of excess ϕES17, no phages were isolated following growth of those colonies in the presence of mitomycin C (data not shown). We concluded that ϕES17 is a lytic phage, lacking an integrase.

#### Host Range of *E. coli* Phages

The host ranges of the phages was determined as described in Section “Materials and Methods” by spot testing serial 10-fold phage dilutions on the isolation strain and on other laboratory and clinical isolates. The virulence of the phage was determined by comparing the titer on a test strain with the titer on the isolation strain (EOP = titer test/titer isolation). Phages with EOP > 0.1 were considered highly virulent and most useful. Phages with EOP in the range of 0.001–0.099, were considered moderately virulent and may be useful if high titers can be produced. Figure 2 shows the host range and virulence results for eight *E. coli* phages on a representative selection of *E. coli* clinical isolates. A complete determination of host range and virulence on (1) characterized ST131 strains, (2) a collection of paired clinical isolates from two sites (urine and blood) collected from the same patient on the same day, and (3) a set of clinical isolates collected between November 2015 and December 2018 is shown (Supplementary Figures S1–S3).

At least one of the *E. coli* phage isolates was able to kill each member of the ST131 collection, except for *E. coli* strain JJ1886 (Supplementary Figures S1–S3). For individual phages, 8–62% of ST131 strains were killed at EOP > 0.1 and 31–69% were killed at EOPs in the range of 0.001–0.099 (Supplementary Figures S1–S3). A cocktail of as few as two *E. coli* phages (ϕHP3 and ϕES17) was capable of killing 12/13 (92%) of ST131 strains (Supplementary Figures S1–S3).

**FIGURE 3:**
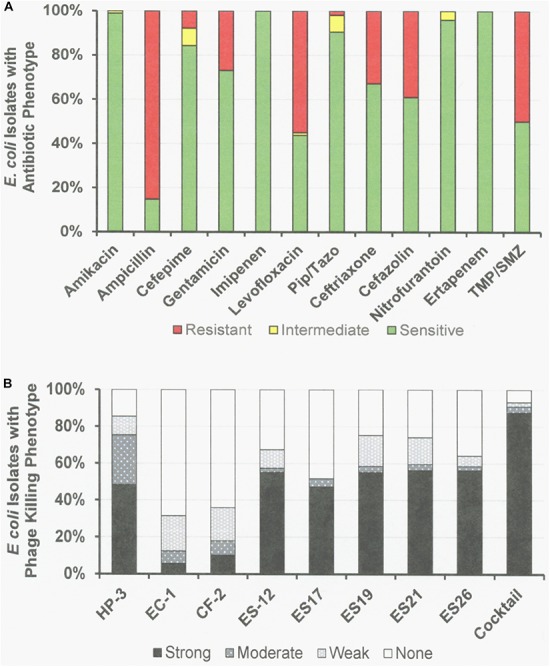
Antibiotic Sensitivities and Phage Killing Phenotypes of *E. coli* Clinical Isolates (*N* = 89). **(A)** Antibiotic sensitvities using cut off values used in the microbiology lab at the Houston VA Hospital. **(B)** The phage killing phenotypes were based on EOP. Strong killers, EOP > 0.1; Moderate Killers, 0.099 > EOP > 0.001; Weak Killers, EOP < 0.00099 but positive; None, no growth. Strong and moderate killers have EOP high enough to be useful in phage therapy. The phage cocktail consisted of equal titers of phages: ϕHP-3, ϕES-12, and ϕES17. Pip/Tazo, piperacillin/tazobactam; TMP/SMX, trimethoprim/sulfamethoxazole.

Among the 76 *E. coli* clinical isolates (24 paired blood and urine isolates from the same patient on the same day; 40 single clinical isolates, mostly from patients with UTI; and 12 clinical isolates from urine of catheterized spinal cord injured [SCI] patients) the eight *E. coli* phages killed from 4 to 57% at an EOP > 0.1. If EOPs between 0.001 and 0.099 (moderately virulent) were included very little increase in the number of clinical isolates killed was observed, except for ϕHP3 where the number killed was increased by 50% (Figure 1). For the 24 paired blood and urine isolates, a cocktail of as few as three of the *E. coli* phages (ϕCF2, ϕES12, and ϕES17) could be assembled that killed them all at EOP > 0.1. Among the 40 clinical isolates primarily of urinary origin, cocktails (ϕHP3, ϕES17, and ϕES19) capable of killing 35/40 (88%) of the isolates at EOP > 0.1 could be made. Among 12 isolates originating from SCI patients, a cocktail of four phages (ϕHP3, ϕEC1, ϕES12, and ϕES17) could be made that killed 9/12 (75%) *E. coli* strains at EOP > 0.1. Among all 76 of the *E. coli* clinical isolates, we noted no correlation between killing at high efficiency and date of isolation (November 2015–December 2018) or antibiotic-sensitivity phenotype. A summary of the antibiotic sensitivity and phage killing of the 89 total E. coli isolates examined is shown in Figure 3. Although none of the individual phages killed more than 50–55% of the 76 bacterial strains at high efficiency, a three phage cocktail increased the high efficiency killing to nearly 90% (Figure 3).

### *Pseudomonas aeruginosa* Phages

#### Origin of *P. aeruginosa* Phages

Four *P. aeruginosa*-specific phages previously isolated and used by other laboratories were used here: ϕKMV (Lavigne et al., 2003; Chibeu et al., 2009), ϕPA2 (ATCC 14203-B1; McVay et al., 2007), ϕPaer4 (Fu et al., 2010), and ϕE2005 (Liao et al., 2012). All four phages as a mixture, or single phages, were used in the host range expansion protocol (HRE) as described (Mapes et al., 2016; see section “Materials and Methods”). The HRE protocol generates phage mutants able to infect and replicate on bacterial strains that were previously resistant to the phage (i.e., broadening their host range). The four parental phages subjected to HRE as a mixture lysed 38% of 16 bacterial strains (development strains) used. After 30 cycles of HRE 75% of the 16 development strains were lysed by the phage mixture. When 10 strains different from those used in the HRE process (test strains) were tested 100% of them were lysed by the phage mixture resulting from 30 HRE cycles (Mapes et al., 2016). During this directed evolution process all phages present are mixed, so that at any cycle the lysate is a heterogeneous mixture of phages. Individual plaques were picked after 20 and 30 cycles of HRE and plaque purified after plating on the desired host. For example, *P. aeruginosa* strain DS38 one of the development strains, was not lysed by the parental phage mixture. The heterogeneous phage mixture from HRE cycle 30 formed plaques on strain DS38 indicating that it contained phages with the host range expanded to DS38. Purified phage clones were generated on strain DS38 from 108 plaques picked from the 30 cycle lysate containing the heterogeneous mixture phages. Among the 108 phage clones that all lysed DS38, there were 30 different killing spectra when they were tested against the 16 development and 10 test strains (Mapes et al., 2016). Similarly, ϕKMV was subjected to five cycles of HRE, and was found have expanded host range phages in the lysate of cycle 5.

#### *P. aeruginosa* Phage Characteristics

**FIGURE 4:**
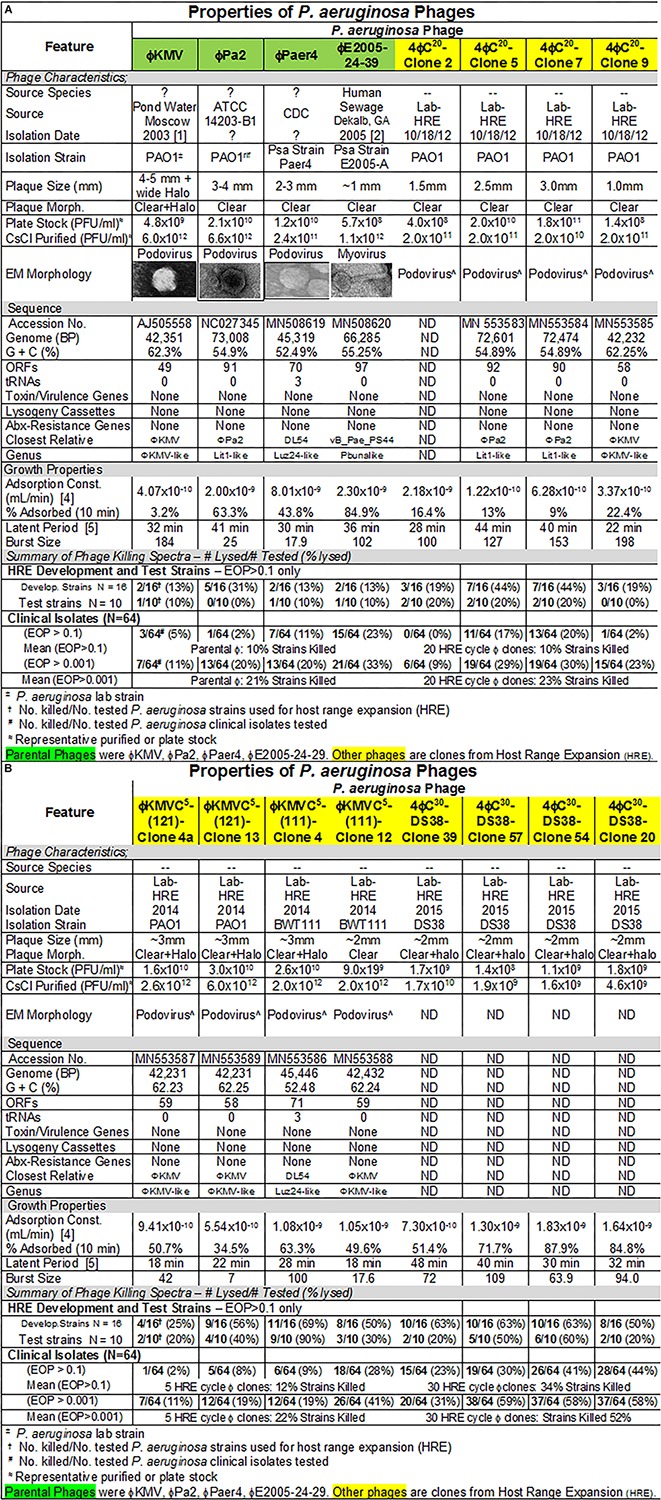
**(A,B)** Summary of characterization of *Pseudomonas aeruginosa* phages. The characteristics, DNA sequences, growth properties and a summary of phage killing spectra are presented for each phage.

The characteristics of parental and host range expanded phages are shown in Figures 4A,B. The characteristics of the phages were variable and similar to those seen for *E. coli* phages (Figure 1). Importantly, DNA sequencing revealed that each of the phages derived from the HRE of the four phage mixture represented only mutants of one of the parental phages. This result indicated that recombination between parental phages did not contribute to expansion of host range in the progeny examined. Thus, the morphology of the HRE-derived clones was not determined but assumed to be like that of the parental phage (Figures 4A,B). Sequencing also revealed that none of the phages contained genes that would be detrimental to their use in phage therapy. The growth properties of the HRE-derived phages were also similar to the parental phages.

#### Host Range of *P. aeruginosa* Phages

**FIGURE 5:**
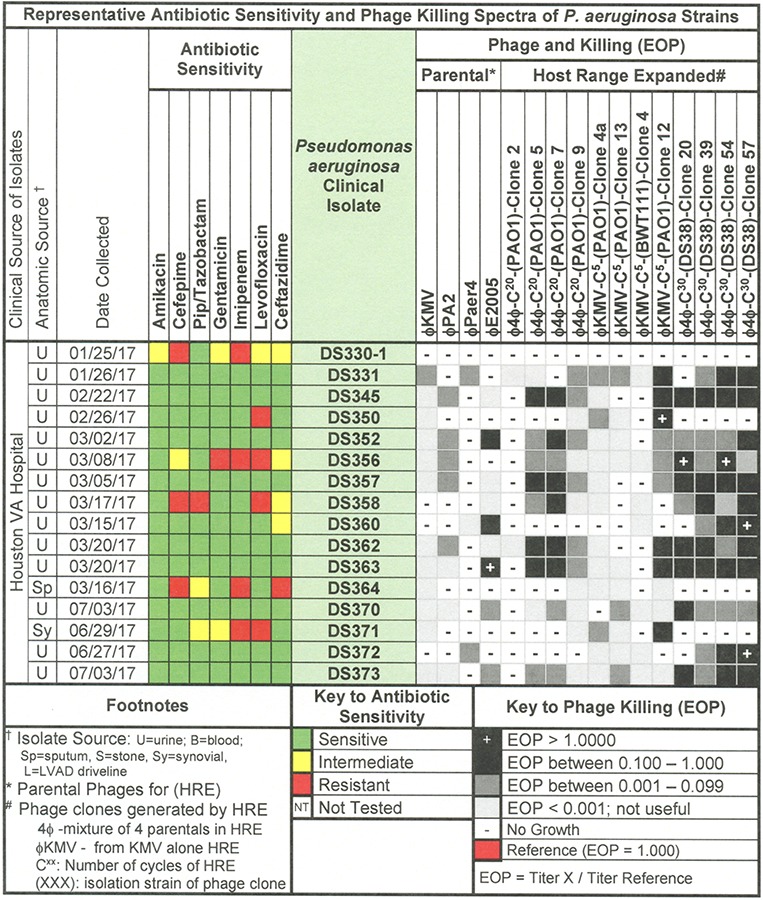
Representative data for antibiotic sensitivity and phage killing (EOP) of clinical isolates of *P. aeruginosa.* Shown are the properties of the *P. aeruginosa* clinical isolates on the left, including: source, date of isolation and antibiotic sensitivity data (VITEK2). On the right are shown the killing spectra of the phages on the individual *P. aeruginosa* clinical isolates. The keys to antibiotic sensitivity and phage killing (EOP) are shown at the bottom of the figure.

**FIGURE 6:**
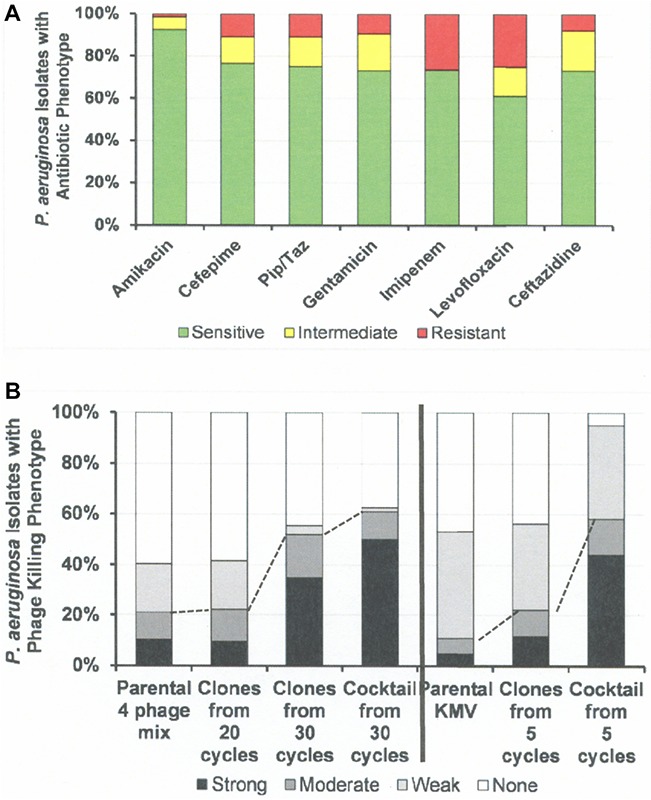
Antibiotic Sensitivities and Phage Killing Phenotypes of *P. aeruginosa* Clinical Isolates (*N* = 64). **(A)** Antibiotic sensitivities based on cut off values used in the microbiology lab at the Houston VA Hospital. **(B)** The phage killing phenotypes were based on EOP. Strong killers, EOP > 0.1; Moderate Killers, 0.099 > EOP > 0.001; Weak Killers, EOP < 0.00099 but positive; None, no growth. Strong and moderate killers have EOP high enough to be useful in phage therapy (dotted lines indicate useful levels of killing). The 30 cycle 4 phage cocktail: consisted of equal titers of the following host range expanded phages: 4ϕC-C^30^-(DS38)-Clone 20, 4ϕC-C^30^-(DS38)-Clone 39, 4ϕC-C^30^-(DS38)-Clone 54, and 4ϕC-C^30^-(DS38)-Clone 57. The “5 cycle KMV cocktail” consisted of clones: ϕKMV-C^5^-(PAO1)-Clone 4a, ϕKMV-C^5^-(PAO1)-Clone 13, ϕKMV-C^5^-(PAO1)-Clone 12, and ϕKMV-C^5^-(BWT111)-Clone 4. Pip/Taz, piperacillin/tazobactam.

Host range and virulence of *P. aeruginosa* phages on representative *P. aeruginosa* clinical isolates is shown in Figure 5. Characterization of the host range and virulence on the complete set of *P. aeruginosa* clinical isolates tested is shown in the supplementary information (Supplementary Figures S4, S5). Compared to the parental phages, the HRE-derived phages had expanded host range when tested against the development strains. They lysed, 19–69% of those strains compared to 12–31% for the parental phages. Likewise the HRE-derived phages had expanded host range when tested against the test strains; they lysed 20–90% of test strains compared to 1–10% for the parental phages (Supplementary Figures S4, S5). The parental and HRE-derived phages were then tested against a collection of 64 clinical isolates, which were mostly isolated from patient urine samples that were obtained between November 2015 and August 2017, and displayed a spectrum of antibiotic resistances. Examination of the killing activity (Supplementary Figures S4, S5) revealed the HRE-derived phages had expanded host range relative to the parental phages, although many of the phages were expanded at EOP < 0.001, an EOP too low to be useful. It is possible that additional rounds of HRE on some the clinical isolates could generate phage with an EOP in a useful range (EOP > 0.001). Greater numbers of HRE cycles led to greater expansion of host range in the isolated phage clones (Figures 5, 6 and Supplementary Figures S4, S5), both at highly efficient killing (EOP > 0.1) and at useful levels of killing (EOP > 0.001). This was observed for both HRE using four parental phages (compare 20 and 30 cycles) and for 5 cycles of HRE using a single parental phage ϕKMV (Figure 5 and Supplementary Figures S4, S5).

Figure 6 summarizes the antibiotic sensitivity and phage killing phenotypes of the phages on the 64 total *P. aeruginosa* clinical isolates examined. The increase in useful killing with cycles of the HRE protocol and with mixing of cocktails is shown in Figure 6B.

The lack of recombination in HRE-derived phages observed here, contrasts with the contribution of recombination reported by others (Burrowes et al., 2019). In retrospect, this finding is not surprising, since the four phages used were distant phylogenetically, making homology-driven recombination unlikely. The HRE-derived phage sequences contained mutations spread randomly across the genome, but all of them had mutations in the tail fiber gene as would be expected if the host range expansion was based on tail fiber-bacterial receptor interactions. In addition, the sequence analysis revealed no genes that would preclude the use of the HRE-derived phages in phage therapy. Thus, the HRE-derived *P. aeruginosa* phages are classified as variants of the parental phage to which they corresponded (Figures 4A,B).

### *Enterobacter cloacae* Phages

#### *E. cloacae* Phage Isolation

**FIGURE 7:**
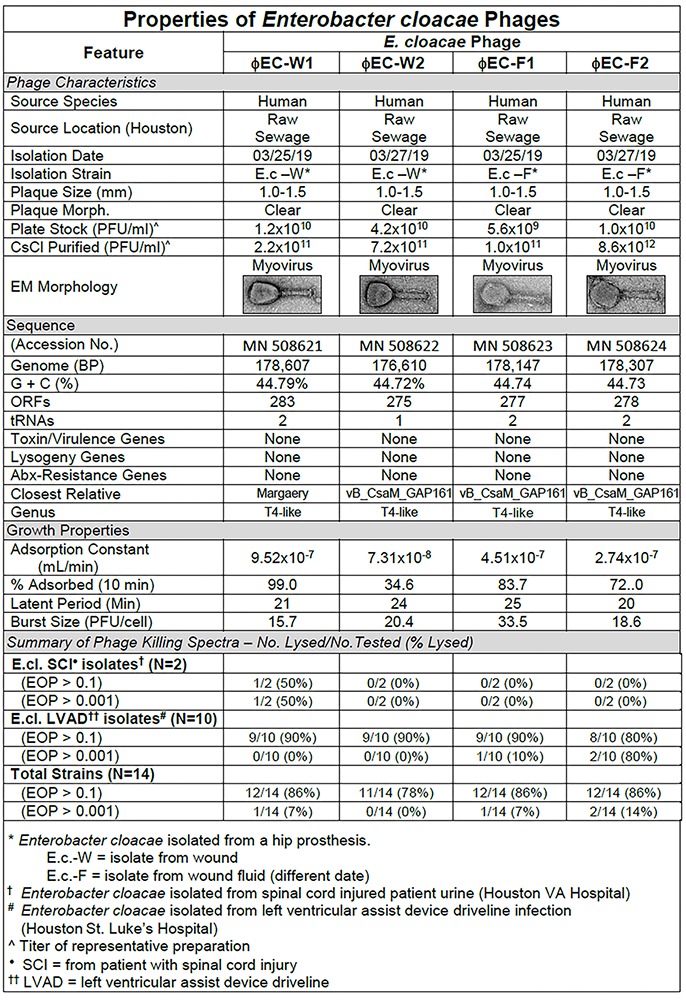
Summary of characterization of *Enterobacter cloacae* phages. The characteristics, DNA sequences, growth properties and a summary of phage killing spectra are presented for each phage.

*Enterobacter cloacae* phages were isolated from raw sewage collected on two different days by plaquing on a phage therapy candidate’s isolates (Figure 7).

#### *E. cloacae* Phage Characteristics

Sequence analysis of the phages revealed that the four phages were similar and T4-like (Figure 7). The growth properties of the *E. cloacae* phages were somewhat variable but had parameters within expected values (Figure 7). The *E. cloacae* phages contained no genes that would preclude their use in phage therapy (Figure 7).

#### *E. cloacae* Phage Host Range and Virulence

**FIGURE 8:**
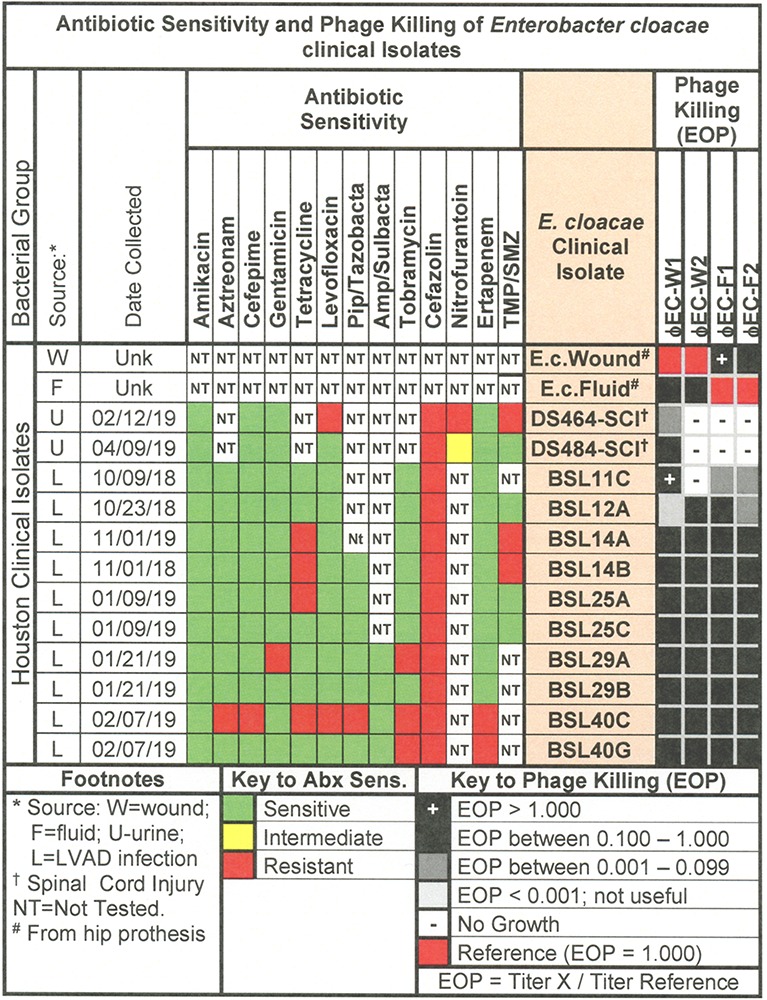
Representative data for antibiotic sensitivity and phage killing (EOP) of clinical isolates of *E. cloacae.* Shown are the properties of the *E. cloacae* clinical isolates on the left, including: source, date of isolation and antibiotic sensitivity data (VITEK2). On the right are shown the killing spectra of the phages on the individual *E. cloacae* clinical isolates. The keys to antibiotic sensitivity and phage killing (EOP) are shown at the bottom of the figure.

**FIGURE 9:**
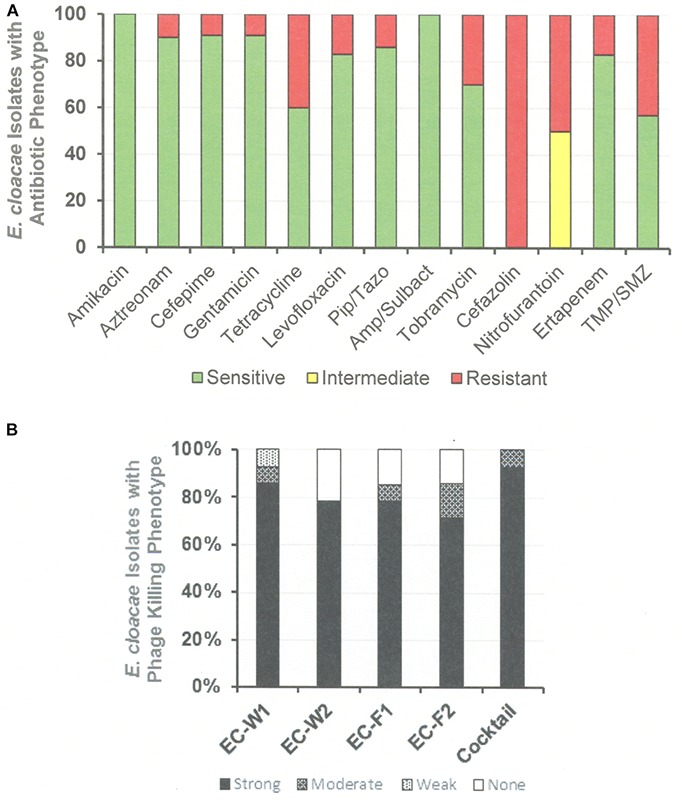
Antibiotic Sensitivities and Phage Killing Phenotypes of *E. cloacae* Clinical Isolates (N-64). **(A)** Antibiotic sensitivities based on cut off values used in the microbiology lab at the Houston VA Hospital. **(B)** The phage killing phenotypes were based on EOP. Strong killers, EOP > 0.1; Moderate Killers, 0.099 > EOP > 0.001; Weak Killers, EOP < 0.00099 but positive; None, no growth. The cocktail was consisted of equal titers of ϕEC-W1 and ϕEC-W2. Pip/Taz, piperacillin/tazobactam; TMA/SMZ, trimethoprim/sulfamethoxazole.

The antibiotic sensitivity and phage killing phenotypes of four phages on *E. cloacae* clinical isolates are shown in Figure 8. The phages were strong killers, especially for *E. cloacae* isolates from LVAD infections where the original source of the infection may have been the skin. Only one phage strongly killed *E. cloacae* isolates from UTI. More isolates from various sites must be examined to determine if site of origin of the bacteria affects the efficacy of phage killing. Figure 9, a summary of antibiotic sensitivity and phage killing of the isolates examined, shows > 70% of strains killed by all individual phages and only a small gain in killing by a cocktail composed of two phages when compared to the best single phage.

## Discussion

This study presents a “*sur mesure*” approach to phage therapy (Pirnay et al., 2011). Here phage libraries were constructed, characterized, and prepared for use in preclinical or clinical situations. Specifically, we plan to concurrently test clinical isolates against phages from the appropriate library to identify phages for mono- or cocktail-based therapy while they are being characterized in the clinical microbiology laboratory. It will then be the physician’s choice to treat the patient with phage alone, antimicrobials alone or the combination of the two. A broader interpretation of the “*sur mesure*” approach is to develop a phage library using the full spectrum of bacterial strains available in the clinical microbiology laboratory of a specific medical facility, so that the shelf-ready phage strains or cocktails can reasonably be expected to cover multidrug-resistant organisms that cause infections in patients in that facility. These libraries can be tested in the clinical laboratory of the hospital, or the hospital can send isolates for phage susceptibility testing to basic science laboratories that agree to participate, such as those associated with educational institutions (Center for Phage Technology, Texas A&M University, United States; The Tailored Antimicrobials and Innovative Laboratories for Phage Research [tialϕr], Baylor College of Medicine, Houston, TX, United States), government laboratories (The Biological Defense Research Directorate of the Naval Medical Research Center, United States; The Eliava Institute, Tbilisi, Georgia; The Phage Therapy Unit, The Hirzfeld Institute, Poland; Center for Innovative Phage Applications and Therapeutics [IPATH], UCSD, San Diego, CA, United States), or industry partners (AmpliPhi Biosciences; Adaptive Phage Therapeutics). For example, we are prospectively collecting bacterial strains from the urine of all hospitalized patients with SCI at our Veterans Affairs Hospital, so that we can create phage libraries to treat any bacterial pathogens causing urosepsis among those SCI patients. Similarly, at the Baylor-St. Luke’s hospital we are collecting all bacterial strains causing infections of left-ventricular assist device (LVAD) drivelines. In both situations our goal is to create a phage library that is able to treat infections caused by the most antibiotic resistant bacteria in that specific clinical setting. While creating phage libraries for many species seems like an attainable goal, it is likely to be more difficult for some species. Phages against *Staphylococcus aureus* are infrequently isolated from environmental samples (Mattila et al., 2015; Latz et al., 2016) and identification of phages active against *Clostridium difficile* required induction of lysogens (Hargreaves et al., 2015). Thus, construction of large libraries of phage will depend on the target bacterial species.

Despite the near certainly that phage-resistant bacteria will emerge during therapy, we envision multiple clinical scenarios in which even a single well-timed dose of phage, in addition to standard antibiotics, may be life saving. For example, rapid initiation of effective antimicrobial therapy is essential to preventing clinical deterioration in sepsis (Kalil et al., 2017). Many patients are at high risk for sepsis caused by antibiotic resistant organisms, by virtue of prior healthcare exposures and/or known colonization with multidrug-resistant organisms. When such high-risk patients present with sepsis, a few early doses of a broad-spectrum phage cocktail used empirically, together with empiric antibiotics could act as a safety net, ensuring adequate coverage of the causative organisms, until the microbiology lab can identify the organism and determine its antibiotic sensitivities. In this scenario a “*sur mesure*” phage cocktail developed against the full panel of multidrug-resistant organisms isolated in the clinical microbiology laboratory of that specific institution would be used for initial treatment together with empiric antibiotics. Another example of a clinical scenario in which a single dose of phage might be very useful would be to temporarily sterilize a patient’s urine prior to an invasive urologic procedure. Phage cocktails mixed specifically for the organisms found in standard pre-procedure urine cultures at a given institution would offer a more targeted approach than our current approach, which involves wiping out the bladder and much of the bowel flora with broad spectrum antibiotics.

In contrast, treatment of biofilm infections, such as those that cause life-threatening LVAD infections, would likely require a longer course of phage therapy, in part because of the longer clinical time frame given the chronicity of LVAD infections. New phage cocktails could be mixed to address phage-resistant bacterial pathogens that might develop during the course of therapy. Alternatively, treating these biofilm infections with phage and antibiotics simultaneously may allow for synergy, particularly if the phage is able to restore antibiotic susceptibility in the infecting pathogen (Comeau et al., 2007; Ryan et al., 2012; Chaudhry et al., 2017). This approach of re-mixing *sur-mesure* phage cocktails and using them together with an antibiotic to which the infecting organism is resistant was successful in treating a patient with disseminated *Acinetobacter* infection (Schooley et al., 2017).

To achieve these clinical goals, we have demonstrated that unmanipulated phages isolated from the environment on *E. coli* ST131, are capable of lysing as many as 58% of a collection of 76 *E. coli* clinical isolates. Cocktails of as few as three of the individual phages (ϕHP3, ϕES12, and ϕES17) were capable of lysing 92% of the 76 clinical isolates. These results indicate that environmental samples provide good reservoirs of phages capable of being used against *E. coli*, and that highly effective cocktails can be generated from them. In all cases the cocktails tested were highly effective against clinical isolates, killing at EOP > 0.1. The highly effective killing of the phages and the high titers obtained in the CsCl-purified preparations indicates that these phages should be useful in clinical therapy where a concentrated dose could be administered without fear of generating a septic response due to the presence of contaminating endotoxin (Figures 1, 4A,B, 7). Similar broad coverage was found for *E. cloacae* phages. In addition, we demonstrated that laboratory isolates of *P. aeruginosa*-specific phages can evolve to expand their host ranges to *P. aeruginosa* clinical isolates. A mixture of four parental phages subjected to 20 or 30 cycles of host range expansion was capable of killing 2–44% of the 64 clinical isolates tested, whereas the uncycled parental phages could lyse only 2–23% of the clinical isolates. However, cocktails containing as few as three individual HRE-derived phages were capable of killing 52 of the 64 clinical isolates tested (81%) (Figure 6). Additional cycles of HRE using the 12 clinical isolates not killed by any of the phages at useable EOP (>0.001), seems likely to further expand the host range among those isolates.

In addition to phage isolation for *E. coli-*, *P. aeruginosa-*, and *E. cloacae*-specific phage collections, a number of parameters were characterized that can be useful in choosing phages for phage therapy, making new phage isolates, and general work with the phages (Abedon, 2017). Our “phage master lists” (Figures 1, 4A,B, 7) contain information on the source of the phages, morphology, growth properties, DNA sequence, and host range much like a Physician’s Desk Reference provides useful parameters for chemical antibiotics. The DNA sequence analyses and morphologies of the phages are important to establish the relationship of the individual phage to other phages in the databases (Weber-Dabrowska et al., 2016; Casey et al., 2018). In addition, DNA sequence analysis provides important information on the properties of the phage genome, ensuring that phages can be used as therapeutic agents because they do not encode genes to establish and maintain lysogeny, toxins, virulence factors, or antibiotic resistance. The data on adsorption constant, adsorption rate, latent period, and burst size all represent parameters that can affect the success of phage therapy (Weber-Dabrowska et al., 2016). Finally, in our determinations of phage host range we examined EOP, a parameter that allows one to determine the relative killing power of a phage on a test strain compared to its killing power on the isolation strain. EOP has been shown to be an excellent method for estimating phage virulence on a given bacterial strain. Simple spot tests of high titer phage were found to overestimate both the virulence and the host range of a phage (Mirzaei and Nilsson, 2015). Indeed, we have shown that phage virulence and bacterial susceptibility to the phage determined *in vitro* allowed us to predict the outcome of therapy *in vivo* (Green et al., 2017). While bacterial receptors for the phages were not identified here, that information is important for the rational mixing of phage cocktails. We are in the process of identifying receptors for phages in our libraries, and have identified the receptor for ϕHP3 as lipopolysaccharide in the *E. coli* JJ2528 host (unpublished data). Having all these parameters at hand aids in the selection of a phage for monotherapy, or a mixture of phages for cocktail therapy. Cesium chloride purified stocks of all phages described here exist and their endotoxin content has been reduced below clinically permissible levels, so that they can quickly be put to use. Our *E. coli*, *P. aeruginosa*, and *E. cloacae* phage libraries are now ready for rigorous *in vivo* studies in animal models of urinary tract infections and LVAD infections, as well as available for compassionate use protocols in humans.

## Data Availability Statement

All datasets generated for this study are included in the article/**Supplementary Material**.

## Ethics Statement

The collection of de-identified clinical isolates was approved by the Baylor College of Medicine Institutional Review Board (IRB).

## Author Contributions

AM, RR, HK, and BT conceived the experiments and guided their performance. SBG, SIG, CL, JC, AT, and KS performed the experiments and analyzed the results. BT arranged for collection of clinical isolates and corresponding antibiotic sensitivity data. RR wrote the manuscript with editing from BT, HK, and AM. All authors contributed to manuscript revision, read and approved the submitted version.

## Conflict of Interest

The authors declare that the research was conducted in the absence of any commercial or financial relationships that could be construed as a potential conflict of interest.
