# Supplementary figures and images for: Constructing and Characterizing Bacteriophage Libraries for Phage Therapy of Human Infections
Source: Front Microbiol. 2019 Nov 12;10:2537. doi: 10.3389/fmicb.2019.02537 (PMC6861333; doi:10.3389/fmicb.2019.02537)

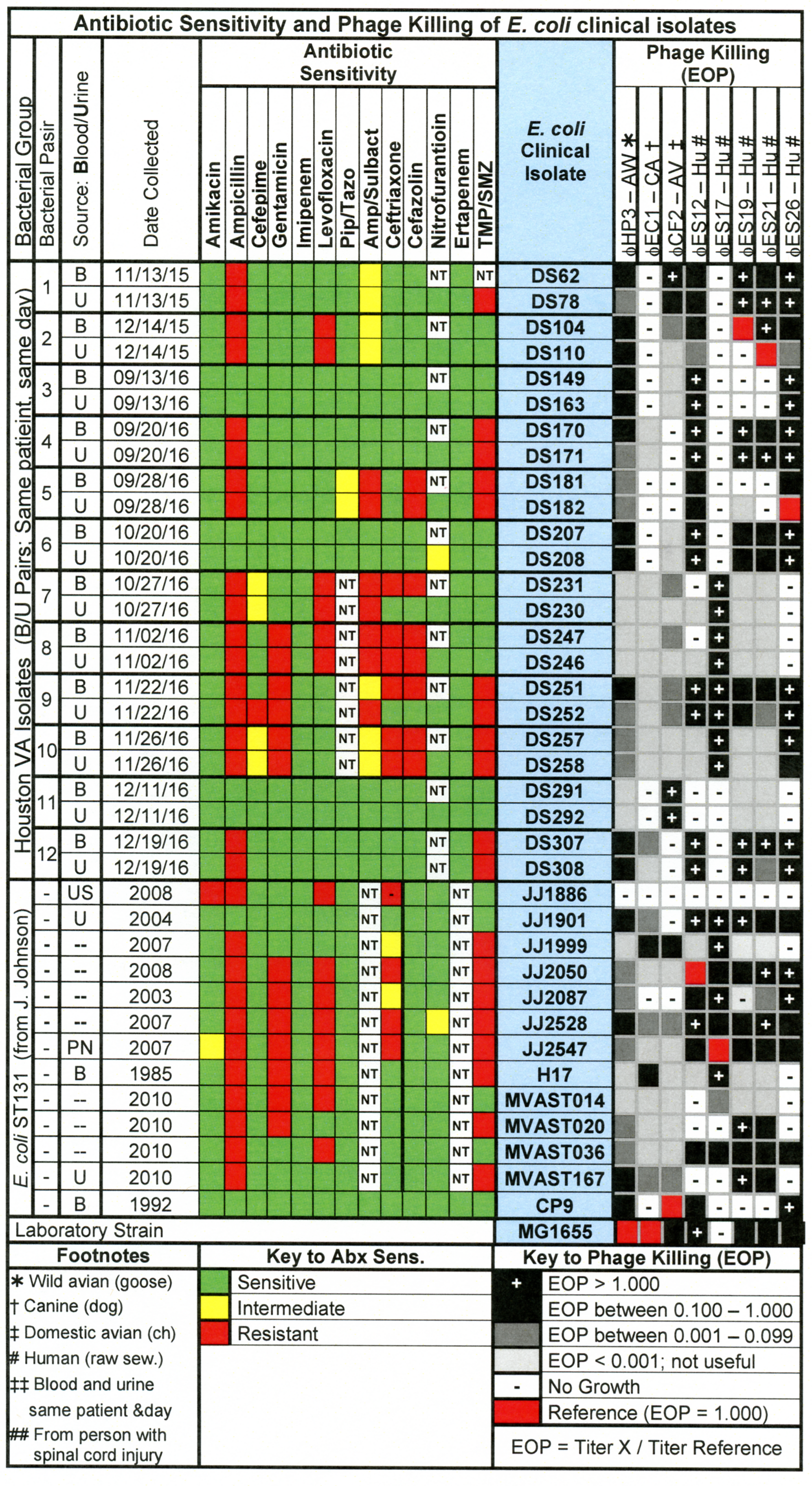

Supplement: Supplementary file 1 [file Image_1.TIF]

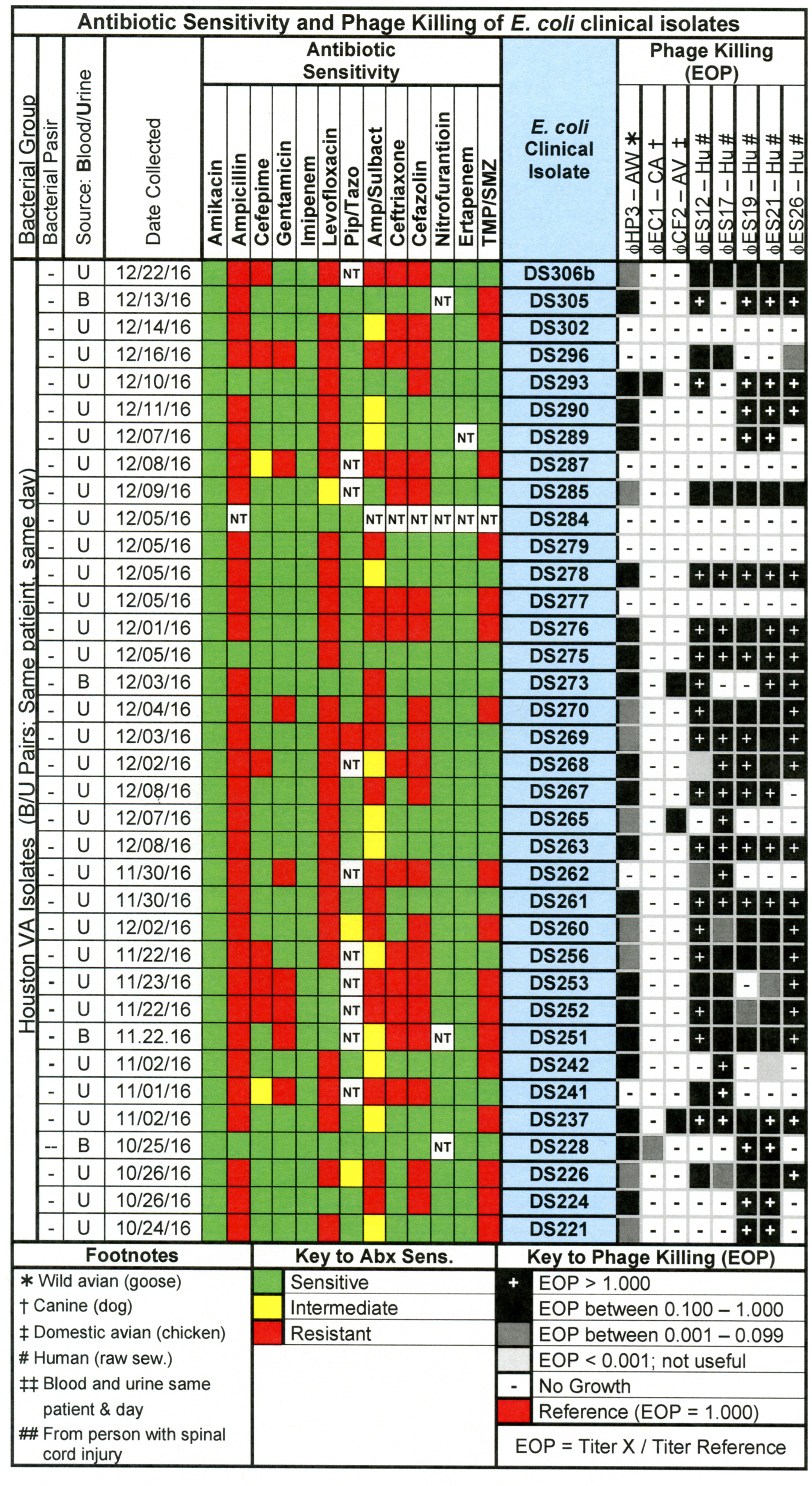

Supplement: Supplementary file 2 [file Image_2.TIF]

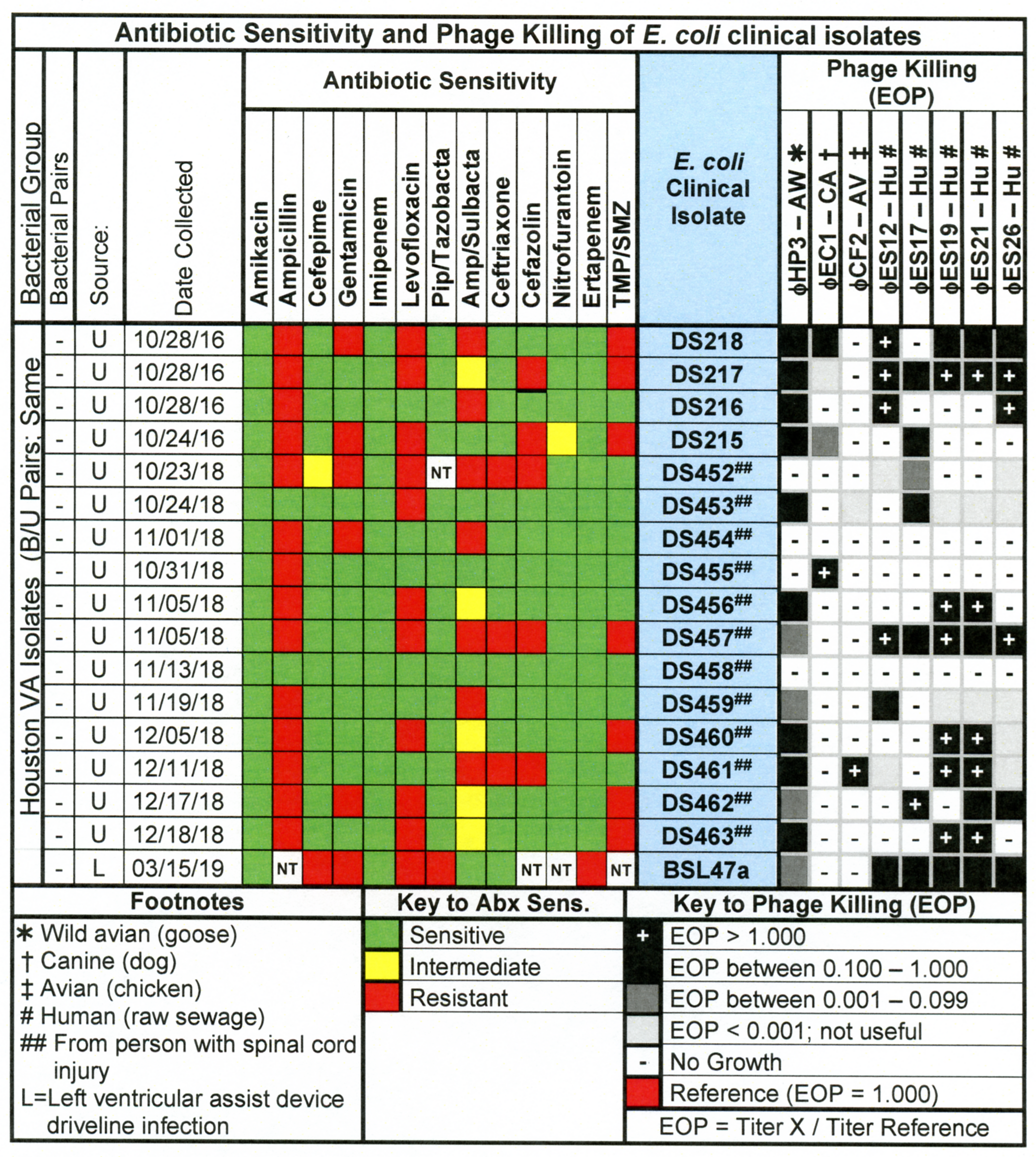

Supplement: Supplementary file 3 [file Image_3.TIF]

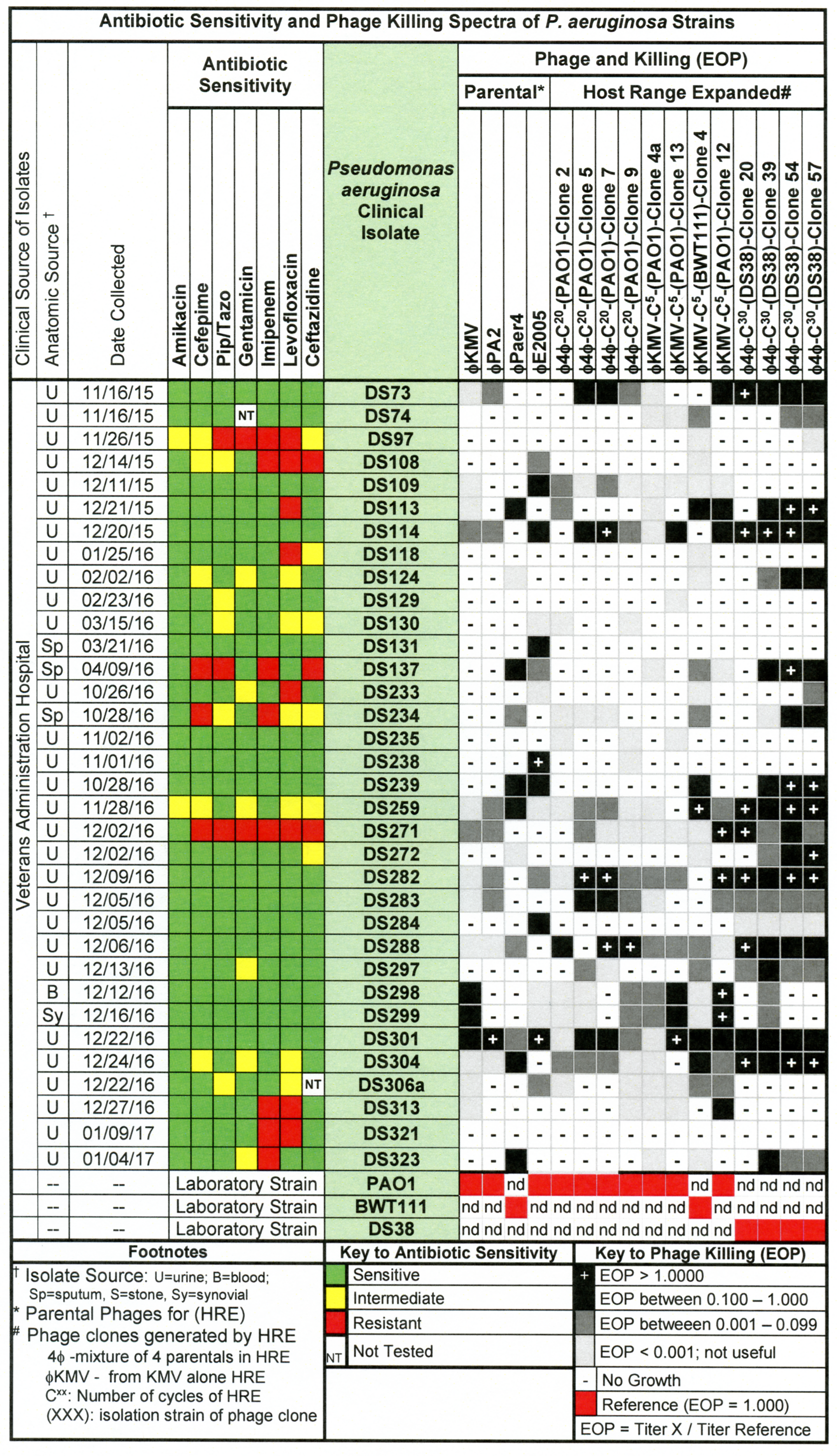

Supplement: Supplementary file 4 [file Image_4.TIF]

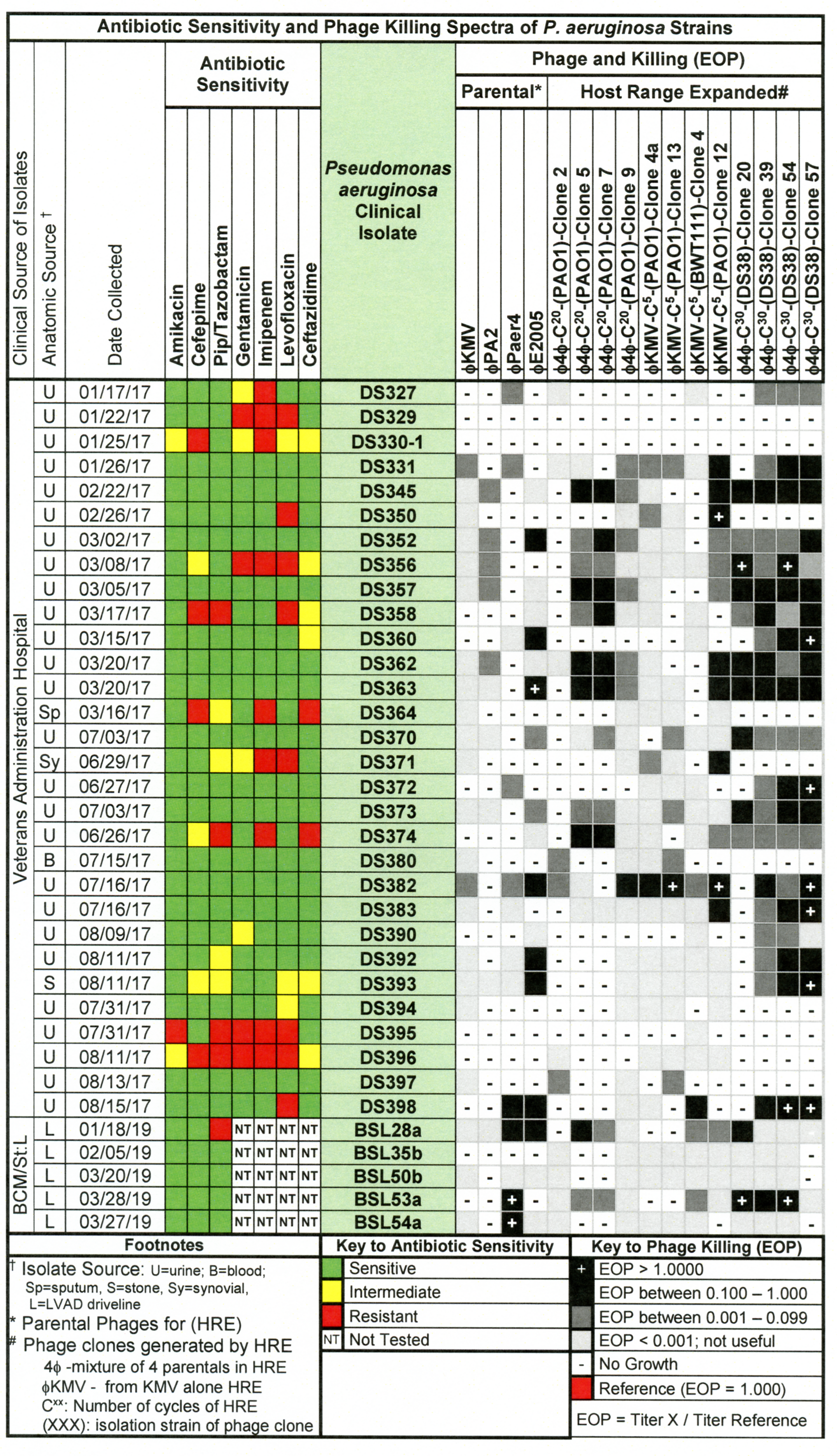

Supplement: Supplementary file 5 [file Image_5.TIF]
